# Primary Neuroendocrine Neoplasms of the Breast: Still Open Issues

**DOI:** 10.3389/fendo.2020.610230

**Published:** 2021-01-26

**Authors:** Marco Gallo, Severo Campione, Valentina Di Vito, Nicoletta Fortunati, Fabio Lo Calzo, Erika Messina, Rosaria Maddalena Ruggeri, Antongiulio Faggiano, Annamaria Anita Livia Colao

**Affiliations:** ^1^ Oncological Endocrinology Unit, Department of Medical Sciences, Città della Salute e della Scienza Hospital, Turin, Italy; ^2^ Endocrinology and Metabolic Diseases Unit, AO SS. Antonio e Biagio e Cesare Arrigo, Alessandria, Italy; ^3^ Section of Pathology, AORN A Cardarelli, Naples, Italy; ^4^ Department of Clinical and Molecular Medicine, “Sapienza” University of Rome, Rome, Italy; ^5^ Endocrinology Unit, Department of Clinical Medicine and Surgery, University “Federico II”, Naples, Italy; ^6^ Department of Clinical and Experimental Medicine, University of Messina, Messina, Italy

**Keywords:** breast neuroendocrine neoplasms, breast carcinoma, review, neuroendocrine, breast cancer

## Abstract

Neuroendocrine breast tumors represent a rare subtype of breast cancer, accounting for less than 1% of all neuroendocrine neoplasms. Starting from their pathology definition, and going through their prevalence, prognosis and treatment, our knowledge is still really uncertain. In the present short review of the medical literature on this topic, we have evaluated in details their epidemiology, risk factors, pathogenesis, pathology, clinical presentation, radiographic aspects, prognosis, and therapy. We have thus been able to identify a number of open issues regarding primary neuroendocrine neoplasms of the breast that need to be clarified. Our ultimate aim was actually to try to understand whether neuroendocrine neoplasms of the breast can be considered a definite clinical entity and if neuroendocrine differentiation of breast tumors has a really clinical relevance.

## Introduction

Neuroendocrine neoplasms (NENs) are a group of heterogeneous tumors deriving from neuroendocrine cells. Neuroendocrine cells are scattered around the body. Therefore, NENs have been reported to arise in multiple sites, such as central nervous system, respiratory tract, larynx, gastrointestinal tract, thyroid, skin, breast, and urogenital system (1).

Primary neuroendocrine neoplasms of the breast (BNEN) are particularly rare, accounting for less than 1% of NENs. Furthermore, the definition of BNEN is still quite confused (2). In the 2003 World Health Organization (WHO) Pathology and Genetics of Tumours of the Breast and Female Genital Organs, BNEN were recognized as a distinct entity (3) requiring -as diagnostic criteria- the expression of NE markers (specifically chromogranin and synaptophysin) in more than 50% of cells (4). It was later revised and the term changed into carcinomas with NE features in the 2012 WHO Classification of Tumours of the Breast (5), with the 50% threshold for NE marker positivity considered arbitrary and therefore removed. In the more recent WHO classification published in 2019, BNEN can only be identified only when the proportion of neuroendocrine cells in samples is greater than 90% (6).

BNEN are overall heterogeneous in their definition, being characterized by a various grade of differentiation and histological overlap, and at present, they do not identify a definite clinical entity, and no specific prognosis or therapy have been recognized yet.

The aim of the present mini-review is to focus on light and shadow of our knowledge about BNEN, and to recognize the gaps to be filled in to better define this group of neoplasms as a precise clinical reality.

## Epidemiology

Primary BNENs represent a rare and heterogeneous entity, whose real incidence is probably poorly understood because they are often under diagnosed (7–10). Its incidence among breast cancer has been reported to range from 0.1% to 5% (5, 9–12). After the WHO classification of breast tumors first recognizing BNEN as a separate unique entity in 2003, Gunhan-Bilgen et al. (2003) and Lopez et al. (2008) analyzed large series of breast cancers, including 1845 and 1368 cases respectively, and estimated that BNEN incidence varied between 0.3% and 0.5% of all breast cancers (11, 12). In 2012 the novel WHO Classification of Tumors of the Breast established that the diagnosis of a NE breast cancer can be made regardless of the percentage of tumor cells expressing NE markers. According to the 2012 WHO classification, primary BNEN would account for 2% to 5% of breast cancers (5, 13). However, Wang et al. analyzed the Surveillance, Epidemiology, and End Results (SEER) registries during 2003–2009 and reported 142 cases of primary BNEN, accounting for no more than 0.1% of total breast cancers, much less than the rate reported by the WHO (9). The relevant changes in the WHO classification criteria for BNEN over the years may explain the large differences in the incidence rate between one study and another. Consequently, further epidemiological studies should be performed by histologically revising tumor samples according to updated and uniform criteria.

Most patients with BNEN are postmenopausal women between their fifth and seventh decade of life (mostly aged >60 years), while the incidence in younger premenopausal women is still lower (5, 9, 14–17). Males are rarely affected by BNEN (9, 15–17). However, despite the overall low incidence, there are proportionally more males with BNEN than with other mammary cancers (9, 17). The distribution of ethnicity for BNEN seems to be similar to other mammary cancers (9).

The main risk factors for BNEN are currently believed to be the same as for non-neuroendocrine breast cancer, such as age and family history. Reproductive factors, as early menarche or late menopause, may also increase the risk of this disease, as well as significant exposure to estrogens, typical of patients taking oral contraceptives or undergoing hormone replacement therapy (HRT) (18). Nevertheless, the risk of breast cancer has been shown to significantly decrease after two years of discontinuation of HRT, as well as in women who stop taking oral contraceptives for more than 10 years (18–20). Evidence exists suggesting a link between high prolactin levels and risk of breast cancer development; however, it is unclear whether this is true for BNEN too. Prolactin has been shown to regulate breast stem cells and progenitor cells through its receptor (PRLR), acting as a pro-tumorigenic pathway (21). Moreover, PRL *via* PRLR is able to promote migration and invasion of breast cancer cells (22). Recently Zang et al. published two cases of BNEN associated with hyperprolactinemia, one patient suffering from mental disorder under antipsychotic drugs, and another one diagnosed with BNEN in late pregnancy, suggesting that hyperprolactinemia may represent a risk factor for the development of BNEN (23).

## Pathogenesis

Current knowledge on natural history and pathogenic mechanisms of breast tumors with NE features is not clearly defined yet. There are different hypotheseson the pathogenesis of these neoplasms. The more controversial one suggests their origin from neoplastic transformation of native neuroendocrine cells constitutively present in the breast, despite little evidence of neuroendocrine cells detectable in benign breast tissue (24, 25). Conversely, according to another assumption, underlying the development of BNEN there would be an early splitting of the neoplastic stem cell differentiation into both neuroendocrine and epithelial lines (26). This assumption is consistent with the typical co-presence of neuroendocrine and exocrine cells in breast poorly differentiated neuroendocrine carcinomas (PD-NECs), and is supported by molecular studies which report a clonal correlation between neuroendocrine cells in PD-NEC and intraductal component of the tumor (25, 27). PD-NECs would therefore seem to originate from a neuroendocrine differentiation of breast cancer cells rather than from endocrine primitive cells.

## Clinical Aspects

Considering the low frequency of BNEN, there is limited knowledge on their specific clinical presentation. There are neither specific clinical signs for this tumor nor differences in the clinical features of primary BNEN compared to other types of breast cancer. Published case series report a slow evolution of these tumors, having as the most frequent reason for consultation being an isolated breast nodule, which is sometimes associated with other local signs (painful axillary adenopathy, bloody nipple discharge, nipple retraction). Clinical presentation with symptoms due to metastatic diffusion (jaundice, hematuria, bone pain, respiratory symptoms, and neuralgia) has also been reported. Less frequently clinical presentations are isolated bloody nipple discharge, isolated skin retraction, anorexia, ulcerated breast masses, carcinomatous mastitis, and Paget like mass (28). In addition, a malignant lesion revealed by routine mammography, performed for family history of breast cancer, has been reported (17). Although extremely rare, a peculiarity of BNEN can be the occurrence of clinical manifestations from ectopic hormonal secretion. Sporadically, case reports of paraneoplastic syndrome with the ectopic production of adrenocorticotropic hormone (29), norepinephrine (30) and calcitonin (31) have been reported.

## Radiology

As well as the clinical features, also the radiological characteristics are unspecific with radiological reports substantially similar to the other malignant breast lesions. Only single case reports (32–35) or small series (11, 17, 36) of BNENhave been reported with their imaging characteristics. Mammographic and ultrasonographic findings of reported BNEN are summarized in **Table 1**. The most common mammographic appearance is a hyperdense, irregularly shaped solitary mass. Margins are more commonly reported as indistinct, microlobulated, or spiculated. In most casescalcifications are absent. Ultrasound evaluation usually shows an irregular or microlobulated hypoechoic lesion, with homogeneous echo texture and no acoustic phenomena. However, posterior acoustic enhancement and, even more rarely, the phenomenon of acoustic shadowing have been reported.

**Table 1:** Mammographic (panel a) and ultrasonographic (panel b) findings in neuroendocrine neoplasms of the breast (BNEN).

Panel a							
Reference	Patients series (nr.)	Location (nr.)	Parenchymal pattern (nr.)	Mass category (nr.)	Margin (nr.)	Shape (nr.)	Calcification (nr.)
Günhan-BilgenI. et al. (11)	CS (5)	UOQ (4); UIQ (1)	fatty (1); heterogeneous density (4)	solitary (4); clustered several nodules (1)	indistinct (1); spiculated (2); microlobulated (1); lobulated (1)	irregular (1); round (4)	absent (5)
Fujimoto Y. et al. (33)	CR	nd	nd	nd	nd	nd	nd
Wu J. et al. (36)	CS (13)	UOQ (5); LIQ (1); UIQ (1); UQ (1); RETRO (2);	scattered fibro glandular density (7); Fatty (2); heterogeneous density (1)	solitary (5); focal asymmetry (12); clustered several nodules (1)	Indistinct (4); microlobulated (3)	irregular (4); round-ovoid (3)	a few, punctuate (1); solitary, punctuate (1); absent (7)
Chang E.D. et al. (32)	CR	UOQ, RETRO	high density	solitary	indistinct	irregular	absent
Yoon Y.S. et al. (34)	CR	LOQ	high density	solitary	Indistinct	round	absent
Hejjane L. et al. (35)	CR (2)	UQ (1); LQ (1); Right (2)	heterogeneous (2)	solitary (2)	irregular (2)	irregular (2)	absent (2)
Ozdirik B. et al. (17)	CS (5)	UOQ (3); LOQ(1); UQ (1) LQ (1)	high density (1)	solitary (1); clustered several nodules (1)	spiculated (1); indistinct (1)	irregular (1); round-ovoid (1)	absent (5)
Panel b							
Reference	Patients series (nr.)		Margins (nr.)	Shape (nr.)	Echogenicity (nr.)	Echo texture (nr.)	Acoustic Phenomena (nr.)
Günhan-BilgenI. et al. (11)	CS (5)		circumscribed (1); microlobulated (2); irregular (2)	irregular (2); round (3)	hypoechoic (5);	homogeneous (4); heterogeneous (1)	none (4); enhancement (1)
Fujimoto Y. et al. (33)	CR		indistinct	irregular	hypoechoic	homogeneous	enhancement
Wu J. et al. (36)	CS (13)		indistinct (4); circumscribed (3); microlobulated (1)	irregular (7); round-ovoid (2);	hypoechoic (9);	homogeneous (4); heterogeneous (5)	none (6); enhancement (2); shadowing (1)
Chang E.D. et al. (32)	CR		lobulated	irregular	hypoechoic	heterogeneous	enhancement
Yoon Y.S. et al. (34)	CR		irregular	round	hypoechoic	homogeneous	enhancement
Hejjane L. et al. (35)	CR (2)		irregular (1);	not reported	hypoechoic (2)	homogenous (2)	none (2)
Ozdirik B. et al. (17)	CS (5)		not reported	not reported	not reported	not reported	not reported

nr, number of cases; nd, not detected; CR, Case Report; CS, Case Series; RETRO, retro areola area; UOQ, upper outer quadrant; LIQ, lower inner quadrant; UIQ, upper inner quadrant; UQ, upper quadrant; LQ, lower quadrant; LOQ, lower outer quadrant.

## Pathology

In the last WHO edition NENs of the breast are classified in neuroendocrine tumors (NETs) and neuroendocrine carcinomas (NECs) (6).

Mammary NETs grossly show clear or infiltrative border, and usually are larger than breast carcinoma of special (ST) or no special type (NST). Histologically, they are composed of large or small nests together with trabeculae, associated with numerous small fine vessels and a variably collagenized stroma, like well differentiated NETs of other organs. Those neoplasms with copious mucous production may also have tumor nests floating in large mucinous pools. Tumor cells range from oval to polygonal or plasmacytoid shape, or also spindled. However, they lack typical nuclear features of NETs, such as salt and pepper chromatin and even monotonous round or oval nuclei, more often displaying irregular appearance or the presence of nucleoli (37).

Diagnosis requires immunohistochemistry (IHC) confirmation by tumor expression of neuroendocrine markers. The most widely used are chromogranin A and synaptophysin. Moreover, the insulinoma associated protein 1 (INSM1) has been shown to have a high sensitivity for NENs arising from different organs, and proposed as an additional valuable marker for BNEN (38, 39).

Breast NENs express hormone receptors (estrogen and progesterone receptors) more often than other breast carcinomas (40). On molecular subtyping, they usually belong to either luminal A or luminal B HER2 negative breast cancer (41–43). Breast NETs are graded in the same manner of breast invasive carcinoma of NST. Differential diagnosis can occur with other primitive mammary tumors that share expression of neuroendocrine markers: solid papillary tumor and mucinous carcinoma. Yet these tumors differ consistently from NETs on morphological grounds to be well separated (44).

Metastases to the breast from NENs of other organs can be distinguished through IHC: Cheratin 7, estrogen (ER) and progesterone receptors (PgR), GATA3 and Gross cystic disease fluid protein 15 (GCDFP15) are quite typical for breast NETs. Moreover, GCDFP15 often shows associated ductal carcinoma in situ, which lack in metastases (45).

Mammary NECs are of the small cell type and morphologically indistinguishable from NECs of other organs. Indeed they show small tumor cells densely packed with basophilic nuclei, scarce cytoplasm, and abundant necrosis. IHC will reveal at least focal positivity for one NE marker. They can express ER, PgR, GATA3, and GCDFP15 (46).

Despite NEC is a very rare neoplasm, its definition and features are clear enough and did never change in the last years. Conversely, NET definition has continuously changed in the last WHO classifications and some controversial issues on its nature still persist (47).

Current WHO classification considers breast NET as the extreme spectrum of mixed neuroendocrine non-neuroendocrine breast neoplasms (MiNeN), in which the non-neuroendocrine component is less than 10%. All mammary tumors in which the neuroendocrine component is between 10% and 90% are breast carcinomas of ST or NST and the neuroendocrine part is only worth mentioning.

## Prognosis

As far as the prognosis of BNEN is concerned, conflicting results have been reported, probably due to the low prevalence of this disease.

In a retrospective study, Lai et al. showed that in a sample of 224 patients with NENs, breast carcinomas expressing high levels of neuroendocrine markers and cytomorphologic features were characterized by a better prognosis. On the other side, a low level of expression of neuroendocrine markers was correlated with more aggressive clinical parameters, such as higher histological grade and pathological T and N stages. These results are suggestive of a better prognosis of invasive breast cancer with neuroendocrine features compared to their non-neuroendocrine counterparts (48). These data were confirmed in a prospective observational study performed on a cohort of 35 patients with BNEN reporting a lower recurrence rate in breast cancers with an expression of neuroendocrine markers >50% compared to those with focal expression (49). These findings provide a stratification based on neuroendocrine markers expression and may be helpful for detecting better treatment strategies, since all invasive breast carcinomas are currently treated according to non-endocrine tumor components guidelines. Furthermore, the study from Lai et al. provides an interesting food for thought, demonstrating a different behavior of CD56-only positive carcinomas compared to those expressing chromogranin and synaptophysin (48). In 2017, Rosen et al. demonstrated a longer overall survival (OS) and disease-free survival (DFS) in patients with BNEN treated with endocrine therapy/radiation therapy rather than chemotherapy, compared to those who did not receive treatment (45). Indeed, no real consensus has been reached on the prognosis for BNEN.

## Therapy

There are no specific guidelines for the treatment of BNEN and the available data mainly descend from anecdotal and case reports. A standardized therapeutic scheme is extremely difficult to define since these are particularly rare and heterogeneous tumors. Currently most of the adopted strategies involve staging and treating BNEN similarly to conventional breast cancer (50), although it is recommended to consider their neuroendocrine origin, especially in the management of well-differentiated tumors (see **Table 2**).

**Table 2:** Overview of therapeutic approaches for neuroendocrine neoplasms of the breast (BNEN).

Treatment		References
Surgery	Breast-conserving surgery/Mastectomy +/- Axillary lymph node dissection +/- Metastasectomy	(2, 51–54)
Radiotherapy	Adjuvant Radiotherapy	(25, 55)
Chemotherapy	Doxorubicin/Cyclophosphamide Carboplatin/Etoposide Cisplatin/Etoposide Fluorouracil/Epirubicin/Cyclophosphamide +/- Docetaxel Paclitaxel +/- Trastuzumab	(17, 34, 35) (55–57) (52) (58–60) (61, 62)
Endocrine therapy	Tamoxifen +/- Aromatase inhibitors	(12, 16, 35, 63)
	Somatostatin Analogues (SSAs) Peptide Receptor Radionuclide Therapy (PRRT)	(64) (65)

The treatment of BNEN is primarily surgical, with partial or total mastectomy in relation to staging and tumor localization (51–54), possibly associated with axillary dissection and/or metastasectomy. Surgery isoften followed by adjuvant radiotherapy, especially for well and moderately differentiated BNEN (25).

Neoadjuvant or adjuvant chemotherapy is widely used in patients with high risk of recurrence or with metastatic or locally invasive disease (25). In clinical practice, well-differentiated BNEN and invasive breast carcinoma with neuroendocrine differentiation (IBC-NED) are usually treated with protocols commonly adopted for conventional breast cancer (eg, epirubicin and cyclophosphamide) and PD-NEC as for small cell lung cancer (eg, carboplatin and etoposide) (17, 52, 55–59, 61). Escape is frequent after a few months, but the neuroendocrine component can be controlled by anthracycline-based therapy (35).

However, a complete response, with a disease free period of thirty-six months after chemotherapy, was recently observed in one patient treated with six FEC 100 (5-fluorouracil, epirubicin, and cyclophosphamide) cycles followed by docetaxel (60). Another rare case of HER-2 positive NECB was treated with trastuzumab with a reported disease free period of 9 years (62).

Treatment with somatostatin analogues (SSAs) has not shown promising results as yet: this is probablyrelated to a non-negligible percentage of BNEN negative for somatostatin receptors (SSR), as well as to theconservative dosages mainly used and to the often advanced disease (64).

The use of peptide receptor radionuclide therapy (PRRT) is currently limited to the treatment of BNEN with somatostatin-receptor (SSR) positivity documented on imaging, after failure of conventional chemotherapy, or even as first-line treatment for advanced disease (65).

Anti-hormonal therapy (eg, aromatase-inhibitors), although not codified, has proven to be effective as adjuvant therapy in some cases of hormonal receptor positive BNEN (12, 35, 63).

Although other agents, such as mTOR inhibitors, are effective for the therapy of different types of NENs, and are also approved, in combination with aromatase-inhibitors, for the treatment of hormone receptor positive advanced breast cancer, currently the use of everolimus has only been hypothesized for BNEN.

Furthermore, Trevisi et al. have quite recently suggested the possibility of target therapy in BNEN, having been reported in a significant percentage of these tumors a mutation of PIK3CA, with alpelisib, as well as target therapy with sacituzumab govitecan targeting TROP-2 protein expressed in a small proportion of BNEN (66).

## Open Issues and Conclusions

Albeit BNEN were firstly described more than 40 years ago, and have been categorized more and more precisely thereafter, its rarity, together with still persisting diagnostic uncertainties, hampers drawing a precise clinical and prognostic picture. Furthermore, the lack of randomized controlled trials performed to compare different treatment strategies and their outcomes makes small case series the best available evidence on this issue, at present (67). Below, some of the main open issues relating to this entity are discussed.

*Histogenesis*—First of all, the same histogenesis of primary BNEN is still uncertain. Nowadays, according to the predominant assumption, they are thought to arise from metaplastic differentiation of a neoplastic epithelial progenitor cell during early carcinogenesis, rather than from a pre-existing neuroendocrine stem cell (53).

*Standardization in neuroendocrine markers use—*Neuroendocrine specific markers are not uniformly and/or routinely applied on every breast cancer sample examined. Only in the event that the pathologist suspects the presence of this type of tumor on histopathological analysis, the sample is subsequently evaluated for the expression of neuroendocrine markers.

Even if this was the case, the lack of consensus on the degree of neuroendocrine differentiation required for the diagnosis limited the uniformity of the diagnostic process, so far. Indeed, all BNEN are combined tumors composed of a heterogeneous mixture of exocrine and endocrine cells. Until 2003, a breast cancer could be classified as a primary neuroendocrine tumor when expressing chromogranin A, chromogranin B, or synaptophysin in more than 50% of the total cell population. Later, differently from the previous WHO classification, diagnosis could be done regardless of a cut-off value of tumor cells positively staining for neuroendocrine markers. Therefore, distinguishing a primary BNEN from a tumor with only partial neuroendocrine differentiation largely depends on the pathologist’s judgement, lacking objective criteria to make diagnosis in case of uncertainties (43, 45, 68).

*Diagnostic criteria*—Consequently, the continuously evolving diagnostic criteria favored the lack of a uniform definition applied by different centers, affecting adequate comparisons and explaining the highly variable prevalence depicted by various case series (68).

Altogether, it is therefore very likely that these tumors are often under-diagnosed/under-reported, in routine pathological practice.

*Prognostic and clinical implications—*Moreover, the clinical and prognostic relevance of neuroendocrine differentiation of breast tumors is still questionable. Conflicting results have been reported on OS and DFS for BNEN vs other types of breast cancer. However, to the best of ourknowledge, none of the already published studies had analyzed primary BNEN based on their distinct histologic or molecular subtypes, rather considering these tumors as a single entity.

Actually, no therapeutic implications apply to the diagnosis of BNEN, and no standardized protocols for treatment of primary BNEN are available. Well-differentiated neuroendocrine breast tumors and invasive breast carcinomas with neuroendocrine differentiations are typically managed with cytotoxic chemotherapy after surgery, similar to more classical types of breast cancer, and poorly-differentiated neuroendocrine breast carcinomas with similar protocols as for small cell lung cancer, whereas hormonal therapy is used according on receptor status.

*Relevance of somatostatin-receptor expression—*Lastly, anecdotal experience concerning the relevance of SSR expression of BNEN for diagnostic (eg, Octreoscan and 68-Ga-DOTATATE PET) and therapeutic (PRRT) purposes represents another main criticism. Presently, PRRT has been recommended for SSR-positive tumors after failure of conventional therapy, but very few reports are available.

Because of all these issues, the discussion of each individual case in an interdisciplinary group of NEN experts is worthwhile in order to provide a tailored treatment for each individual patient (17).

In conclusion, it is likely that the cases diagnosed as primary BNEN are only minimally representative of their real prevalence. Only a systematic evaluation of neuroendocrine markers on all analyzed cases of breast cancer could give a reliable evaluation of the frequency of these tumors. Cancer registries centralizing uniform data collection, together with large multicentric studies, could sharpen our knowledge in the next future.

On the other hand, larger histologic and molecular-profiling studies of this rare but often under-reported cancer, with correlation with clinical data, would be warranted to shed light on BNEN. It is also possible that, at the end of these efforts, we will come to the conclusion that neuroendocrine features poorly add (if any) to the diagnosis and management of breast cancer, but the time has come to conclusively define the clinical relevance of neuroendocrine differentiation of breast tumors.

## Author Contributions

All authors have contributed equally to the conception and design of the review. MG, AF, and AC conceived the review. MG, SC, VV, NF, FC, EM, and RR reviewed published literature and drafted the article. AF and AC revised the manuscript critically. All authors contributed to the article and approved the submitted version.

## Conflict of Interest

The authors declare that the research was conducted in the absence of any commercial or financial relationships that could be construed as a potential conflict of interest.
